# Super Champions, Champions, and Almosts: Important Differences and Commonalities on the Rocky Road

**DOI:** 10.3389/fpsyg.2015.02009

**Published:** 2016-01-11

**Authors:** Dave Collins, Áine MacNamara, Neil McCarthy

**Affiliations:** ^1^Institute of Coaching and Performance, University of Central Lancashire, PrestonUK; ^2^Gloucester Rugby Club, GloucesterUK

**Keywords:** psychological characteristics, developmental growth, coping, non-linearity, challenge

## Abstract

The real-world experiences of young athletes follow a non-linear and dynamic trajectory and there is growing recognition that facing and overcoming a degree of challenge is desirable for aspiring elites and as such, should be recognized and employed. However, there are some misunderstandings of this “talent needs trauma” perspective with some research focusing excessively or incorrectly on the incidence of life and sport challenge as a feature of effective talent development. The objective of the study was to examine what factors associated with such “trauma” experiences may or may not discriminate between high, medium, and low achievers in sport, classified as super-champions, champions or almosts. A series of retrospective interviews were used with matched triads (i.e., super-champions, champions, or almosts) of performers (*N* = 54) from different sports. Data collection was organized in three phases. In the first phase, a graphic time line of each performer’s career was developed. The second phase explored the specific issues highlighted by each participant in a chronological sequence. The third phase was a retrospective reflection on “traumatic” motivators, coach/significant other inputs and psychological challenges experienced and skills employed. Data suggested qualitative differences between categories of performers, relating to several perceptual and experiential features of their development. No evidence was found for the necessity of major trauma as a feature of development. There was a lack of discrimination across categories of performers associated with the incidence of trauma and, more particularly, life or non-sport trauma. These findings suggest that differences between levels of adult achievement relate more to what performers *bring to* the challenges than *what* they experience. A periodized and progressive set of challenge, preceded and associated with specific skill development, would seem to offer the best pathway to success for the majority.

## Introduction

There is considerable evidence that the real-world experiences of young athletes follow a non-linear and dynamic trajectory (Abbott et al., 2005; Ollis et al., 2006; MacNamara et al., 2010a,b; Bridge and Toms, 2012). In contrast to the deliberate practice framework (Ericsson et al., 1993) that suggests expertise is a linear function of time spent in practice, successful athletes typically report a non-linear, complex, and individualized route to the top of their sport and must adapt to (anticipated and non-anticipated) developmental opportunities, set-backs and a range of transitions as they progress in their sport. In an effort to understand, and support, the development of young athletes it is therefore important to understand the nature of this pathway and the range of factors that may positively, or negatively, impact on development and distinguish between those athletes who achieve the greatest success and their less successful counterparts.

A common feature of many talent development (TD) pathways is to minimize the number and certainly the impact of developmental challenges on young athletes. For example, providing young athletes with financial, coaching, and sport science support in a supportive environment is a common feature of TD pathways. This approach is undertaken in an effort to minimize challenge and allow young athletes focus on their sporting commitments. Indeed, many TD pathways purposefully try to smooth the pathway for their most talented performers. The problem is further exacerbated since, due to their early ability, many young athletes may often not encounter many challenges until late in their career. Increasingly, however, experiencing a degree of challenge during development is recognized as an essential characteristic of those athletes who make it to the very highest level in their sport. As such, there has been calls for the inclusion of structured trauma, designed and implemented as part of the pathway, as an essential feature of any TD system (Collins and MacNamara, 2012). There seems to be growing recognition that facing and overcoming a *degree* of challenge is desirable for aspiring elites and as such, should be recognized and employed, rather than avoided. Of course, addressing terminology in this area is important. In fact, the use of the term ‘trauma’ often seems to raise some concerns due perhaps to the emotive nature of the word. An Oxford English Dictionary definition of trauma would generate the following “from Greek, literally wound”; in short, anything from a small cut to losing a limb! We would contest that the use of trauma in the TD context is certainly more toward the band aid than the amputation end of the continuum, albeit that, at the time, the emotional upheaval from the trauma can be very real for individuals. For young athletes on the TD pathway, this structured trauma might include challenges such as playing up an age-group, out of position, de-selection or selection for particular competitions, or increases in training load (Collins and MacNamara, 2012).

It is also important to consider the skills required to negotiate this “challenge-filled” pathway. Considerable research has highlighted the importance of psychological characteristics and competencies as central to the development process. For example, our recent work in TD has identified a set of skills which can both facilitate the process and optimize the outcome of the talent pathway. Termed the Psychological Characteristics of Developing Excellence (PCDEs: MacNamara et al., 2010a,b), and described as those skills and characteristics that enable young athletes cope with the inevitable “up and downs” of development, maximize growth opportunities, and learn from setbacks, we see these characteristics as an implicit element for development and exploitation throughout the pathway. Having identified them, the next logical step was to look at how these characteristics were optimally developed and deployed (cf. Gould et al., 2010). Based on our studies and the wider literature, there seemed to be two important sources of PCDE-like characteristics, apart from specific interventions focused on their development (e.g., MacNamara, 2011).

The first was the background and life experiences of the performer; for example, issues such as aspects of upbringing (e.g., status as an ethnic minority) seem to be associated with eventual sporting success (e.g., Van Yperen, 2009). The second were experiences in the pathway itself (cf. Nicholls et al., 2006; MacNamara et al., 2008). For example, there appears to be considerable merit in the teaching of PCDEs and offering opportunities to test and refine these skills against real-life challenges. As such, the explicit teaching of PCDEs, exercised and supported against developmental challenges along the pathway has been shown to equip athletes with the generalizable skills, and how to deploy them appropriately, to cope with ‘trauma’ (Collins and MacNamara, 2012). Both these sources pointed toward a non-linear (cf. Abbott et al., 2005) and challenge filled progression along the talent pathway as a common characteristic of high-level participants (e.g., MacNamara et al., 2010b). Accordingly, and building on these earlier data, we proposed the Rocky Road to Success (Collins and MacNamara, 2012), highlighting the incidence of challenging events as key defining moments in elite performers’ autobiographical accounts of development and in addition, the increasing recognition of psychological factors as central to all aspects of performance (e.g., Duckworth et al., 2007, 2010). In summary, we proposed that talent *needs* some degree of challenge to develop optimally and thus, talent pathways had to optimize challenge rather than merely providing unremitting support. Importantly, ‘challenge’ episodes should be preceded by skills training, and fully supported and debriefed, if the benefits of this approach are to accrue (Collins and MacNamara, 2012).

Notably, however, although such “bumpy,” challenge-filled pathways were a feature of every high level performer we examined, independent of domain (MacNamara et al., 2010b), there were several aspects of this pathway experience and its antecedents which needed exploration. Specifically, and reflecting the terminological confusion highlighted previously; trauma, challenge, and “rocky road” have all been used to describe the growth episodes described in this research field (e.g., Collins and MacNamara, 2012; Howells and Fletcher, 2015) and there was a clear need, from both a research and applied perspective, to provide clarity. Furthermore, several authors have pursued research lines which appeared to suggest that it is such trauma itself which is the causative factor in high sporting achievement. Typical approaches examined athlete biographies and autobiographies, highlighting the number and nature of occurrences which had occurred in the lives of top performers (e.g., Oakley, 2014; Howells and Fletcher, 2015). There are even some sources suggesting the juxtaposition of trauma and early sporting achievement as a consistent or even exclusive precursor of later elite multiple success (Rees et al., 2013). This seems to us as practitioners to be unlikely and, as a proposed evidence base for TD policy in sport, offers little for the development of pre-elites. Instead, carefully structured, well-supported and individualized challenge as an essential feature of the TDE would seem more developmentally appropriate.

Accordingly, we were interested to see what factors associated with “challenge” experiences may or may not discriminate between high, medium and low achievers in sport. Therefore, we conducted interviews with matched triads in an attempt to tease out differences and similarities within their developmental experiences.

## Materials and Methods

### Participants

Given the aims of the study, and with particular reference to the groupings identified in the title, we purposefully sampled triads of performers from different sports. Each triad was matched as closely as possible on basic background characteristics (i.e., sex, years in sport, sport/event/position played, nationality, educational level) to facilitate comparison.

We targeted and recruited from four groups of sports to offer a range of experience. Accordingly, 54 performers were selected and interviewed as follows; six triads from team sports (18 participants from soccer and rugby), six triads from CGS (centimeter, gram, second) sports (cf. Moesch et al., 2011 – 18 participants drawn from athletics and rowing/sculling), three triads from individual sports (curling, shooting, and skiing) and three from combat sports (karate, judo, and boxing). Participant confidentiality was clearly a paramount concern, so demographic details are not reported or approximated. Furthermore, in order to secure a sufficiently “super”, super champion category, we recruited from several Western European nations rather than just one. Control for differences in national experience was achieved by recruiting each triad from a single country and, where possible, ethnic group.

Participants were classified on the following criteria. In team sports, super champions (SCs; see **Table 1**) had all played/were playing premiership level (i.e., English Premiership – Rugby Union; English premier league, Bundesliga, or La Liga – Soccer) and had achieved, at the time of interview, more than 50 appearances for their national team. Champions (Cs; see **Table 1**) had also played/were playing at the same league level but had achieved less than five international caps. Almosts (As) were defined as players who had achieved well at youth level (including age-group representative honors) but had then played/were playing at Championship (second national league) as their highest achievement.

**Table 1:** Participant achievement information.

	Super champs	Champs
Team	73.4 caps	4.2 caps
	22.5 years	12 years
	Four still playing	Three still playing
Individual Sports	8.25 Medals	1 Medal
	13.2 years	12.3 years
	Four still competing	Two still competing

In all the other sports, we used a combination of time at high levels of world ranking in combination with major (world, Olympic) medals won as the classifier for SCs (see **Table 1**). This facilitated balance of achievement across differing sports and avoided issues of those sports with multiple rather than single/double medal opportunities at single events (e.g., cycling/swimming versus athletics, respectively). Accordingly, SCs had achieved ranking/were currently ranked in the top 3 in the world for their event for at least four years *and* had won at least five world/Olympic medals (or the equivalent in professional/non-Olympic sports, e.g., World-Cup), with no more than two achieved at the same event (to ensure that the SC status had some longevity). Cs (see **Table 1**) had been ranked/were ranked in the world top 40, achieving no more than one medal at world or Olympic level. Finally, As were classified by their achievement of world/European youth/junior medals, but no medaling performances at this level as seniors. In all cases, across all sports, performers were either still active or had retired within the last 3 years when interviewed.

### Procedure

Participants meeting the criteria outlined above were invited to participate through personal contact, either directly or through gatekeepers. Fifty-six participants agreed to take part and completed informed consent, the study having been approved through the University Ethics Committee.

**FIGURE 1:**
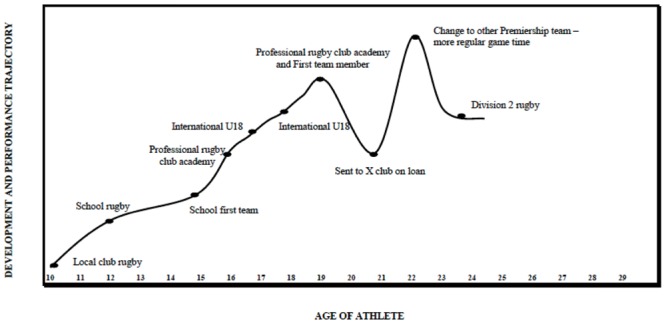
**Exemplar participant drawn timeline (Team Sport Almost) of development pathway**.

Data collection was organized in three phases using a semi-structured interview method. To ensure an optimum personal context to the data, it was crucial that each participant was able to relate his or her experiences clearly to the key stages that applied to their own careers (cf. Ollis et al., 2006). This approach has been previously shown to increase the accuracy and veracity of recall (Drasch and Matthes, 2013) and was an important step to help overcome some of the limitations of retrospective recall inherent in this type of data collection by ensuring participants anchored their recall of incidents to particular times and events. Accordingly, in the first phase and in collaboration with each participant, a trajectory chart of each performer’s career was developed on a standardized grid (see **Figure 1**). Guided questioning, using a standardized interview guide including probes and prompts, enabled an exploration of the different stages encountered along the pathway to excellence which included both sport and non-sport related events.

Building on this stage of questioning, the second phase explored the specific issues identified in the first phase of questioning, following a chronological sequence from pre-pathway, then during the macro and micro stages and transitions identified in the first part of the interview. Again, a standardized interview guide including probes and prompts was employed with each participant and all interviews were conducted independently. Finally, the third phase addressed a retrospective reflection on “traumatic” motivators and derailers, coach/significant other inputs, methods employed (including PCDEs) and psychological challenges experienced. Each question in the interview guide was open-ended, thus yielding a variety of responses that were pertinent to each performer. Specific probes and prompts were used for clarification and elaboration of key points and to obtain consistency in the depth of responses (Patton, 2002).

### Design and Analysis

All interviews were transcribed verbatim with each interview lasting between 90 and 130 min. Following this, and using the self-drawn trajectory charts, participants’ experiences were tracked across the pathway process. Drawing on these retrospectives, plus participants’ *post hoc* viewpoints, inductive content analyses were conducted. Specifically, after reading and re-reading the transcription, qualitative analysis software (QSR NVIVO 9) was used to transform raw data units into thematic hierarchies by recursively engaging in tag creation, category creation, and category organization (Côté et al., 1993). As the first step, a line-by-line analysis was undertaken to identify and label raw data units (i.e., raw quotation that exemplified a meaningful point or piece of information). Following this, the raw data units were compared for similarities and grouped into higher order themes. To revise identified concepts based upon emerging analysis, the constant comparative method was employed and conceptual memos recorded detailing evolving ideas and key notes (Davis and Meyer, 2009). This process allowed for the constant refinement of the results until theoretical saturation was met (Strauss and Corbin, 1998). Reflecting the focus on common themes in experience, data were then amalgamated to produce a set of perceptions indicative of the group as a whole.

#### Addressing Trustworthiness

Several approaches were employed to optimize data trustworthiness (cf. Sparkes, 1998; Sparkes and Smith, 2009). All interviews were conducted by the first author who enhanced trust and rapport with interviewees via appreciation of their history and current situation and the demands of the general/specific elite performance and development experiences. In addition, interview transcripts were returned to each participant to allow member checks with participants, involving a 10–15 min meeting/skype conversation to discuss the emerging results and the accuracy and fairness of quotes considered for inclusion in the paper from that individual. Importantly, feedback was sought on what the researchers had considered these quotes to signify and the context of the results subsection in which they would appear. From this process, no thematic categories were changed and four of 60 exemplar quotes were slightly adjusted without altering their meaning.

Trustworthiness of the analytical process was also addressed. Facilitated by QSR NVIVO’s optimization of transparency (cf. Bringer et al., 2004), the constant comparison method and creation of conceptual memos (Davis and Meyer, 2009) as well as challenging data interpretation ensured that evolving meaning was continually re-evaluated and re-asserted. To further limit interpretative bias and verify rigor, a reflexive journal was also maintained (Patton, 2002). Additionally, the third author (an experienced qualitative researcher) read the full transcripts of nine interviews and assessed *a priori* interpretations of meaning units against the labels created by the first author and their fit with the overall thematic structure. In the few cases of alternative explanation and questions over accuracy or potential bias, reflective discussion took place until all themes and their location in the thematic hierarchy were agreed (Stake, 2008). Furthermore, and also ensuring that the first and third authors’ remained cognizant of their assumptions and presumptions, the second author acted as a “critical friend” throughout by supporting in-depth critique and investigation of the emerging interpretation, discoveries, and explanations (Faulkner and Sparkes, 1999).

## Results

Reflecting the stated aim of the investigation, we highlight those factors which did, and then did not, discriminate between the different categories of performer. We should highlight that only rarely were these differences objective, depending rather on qualitative factors such as reported experience, perceptions, etc. To offer a rich picture of the data, exemplar quotes are presented in each section, which represent the clearest themes to emerge from the data.

### Discriminating Factors

#### Commitment

Interest in and commitment to their eventual sport characterized all the SCs, even though they did maintain other activities during their development years. This was a consistent factor for the SCs across all the sporting categories, as highlighted by the following examples:

To be honest the only other sport that I really did was basketball. And that was round about the age of 13. I wasn’t very good at it though so I lost interest quite quickly. I loved doing athletics, I really enjoyed it, and I did sports at school through PE lessons but there nothing that really kind of interested me outside of athletics. [CGS-SC]

I did athletics, handball and skiing but football was always king… like skiing for example I didn’t do competitions I practiced and trained. Also I was doing really well in high jump as a youngster, at 13 I competed at like nationally but I never really trained. [Team-SC]

The only differences apparent in the SC category were where participants were focused initially on another sport.

I love [team sport]! I did everything at primary school, my PE teacher introduced [team sport] to us. She didn’t really know it very well, she was teaching us out of a book! So she was standing and reading ∗laughs∗ but I just loved it. I don’t know why, I think it was just a very new sport, a very novel sport compared to the other sports I played. And yeah, I was just hooked on it from then. [CGS-SC]

A similar early profile was apparent in the majority of Cs. For example, one of the team sport Cs described how from a young age:

…all I wanted to do was play all the time and I knew that was what I wanted to do when I was older from a very young age. I was always kind of the most committed, I would miss out on going to parties on Friday or Saturday nights because I knew I would be playing…yeah I was pretty disciplined as a young lad. [Team-C]

There were some notable differences, however, which extended toward the transitional period.

I played every sport but I smoked when I was younger from the age of 14 to 18 and I mean everything was blasé and laid back and a quite immature approach to it and nothing really mattered because it didn’t need to. [CGS-C]

In contrast, As were often less committed: whilst all reported loving their sport, playing was often more important than training. Typifying this, a number of the team As described how:

I was probably up to that point coasting. Because you were playing every week you sort of take it for granted that you will be in the team because you were top of the pile. [Team-A]

Regularly playing A league and playing against those guys and training on a regular basis and just the odd session with the first team training on a regular basis. I felt comfortable doing that, it was not like it was a shock. [Team-A]

This approach and attitude to competing, rather than training, was further highlighted by one of the combat As:

I loved fighting, but the training was just a chore. I would miss it if I could and always avoided the bits I was shit at. [Combat – A]

#### Reaction to Challenge

Super champions were characterized by an almost fanatical reaction to challenge, both proactively and in reaction to mishaps (i.e., trauma) which typically occurred due to injury or sport related setbacks such as non-selection/being dropped. For example, one of the team sport SCs described how:

I always felt that there’s no chance, nobody or anybody could train more than I did. I always had that confidence. Even though I was a shy person I had that confidence in knowing that I must have been training at least as much as those who are training in a bigger club. [Team-SC]

In a similar vein, another team sport SC explained how his reaction to challenging events was in fact a positive developmental experience:

I think I was quite critical to myself and I felt um…, I was too critical sometimes. But um… when you look back at it, it just did me good because I continued to develop but I felt like I could play one or two bad match then the third I was playing really well, this is how I felt it was going. [Team-SC]

This positive reaction to challenge was typified by a “never satisfied” attitude as described by both of these SCs:

I was never kind of satisfied, I was never like ‘Oh I’ve done it now’ I was always like ‘This is the first step of my journey’ and there’s worlds, there’s Europeans and that brings with it tougher competition. [CGS-SC]

My mum said I was never satisfied; I always wanted to do something else, always planning. [Combat-SC]

In fact, this internal drive was apparent across sports, with SC performers driven from an early age.

I started a lot further back than a lot of other girls. So I was always really proud of that, how I’d manage to drag myself up to be a good enough person to be selected. I knew I wasn’t as good as everyone else but the fact that they saw so much potential. [CGS-SC]

I am very much the sort of person that if I am not progressing or not improving in an area I will get frustrated. So whatever the activity, I was always wanting that next step…once I felt that I had taken a step and that kind of renewed my energy and my passion for it, and again at the bottom of another ladder. [Individual-SC]

Most significant, however, was the super champs’ reaction to setback. They spoke about how setbacks, injury or deselection for example, were catalysts for their development rather than roadblocks:

That injury was pretty crucial I think…I was going well before it but the disappointment…the pain…it just kicked me where it hurt and I was determined to get back. [Combat –SC]

Not making that selection, especially after all that work. Several others just said fuck it, but I was never ever going to let them beat me. I just did double everything! [Individual-SC]

The formative nature of these challenges is particularly apparent in this quote from a SC who suffered a potentially career ending injury.

No never, never ever thought about giving up. There was days when I was like ‘Why is this happening to me? I’m so frustrated, what am I going to do? How long is it going to take me to get back?’ But then the other days were like, ‘right what do I need to do? I’m going to do this, do this and get back’. But I never ever thought I wanted to quit. I think I still would have worked hard and still trained and done everything I could have done. But I think it gave me a different mental capacity. Because I’d never had to deal with anything like that before, so I definitely did think it changed me and made me achieve what I then went onto achieve. [CGS-SC]

By contrast, Cs displayed a much less consistent drive.

Rather than staying at training and thinking ‘right I’m going to work hard, I’m going to really focus on my crossing, or really focus on that’, I did no extra work. I didn’t go in the gym, I didn’t eat the best foods. [Team-C]

Skill gaps were often ignored, with the performer making up this deficiency through effort rather than working at it as a significant weakness.

I played hooker in the three-man scrums so was one of the smallest but I was probably one of the hardest working in terms of the mental side of it. Skill-wise I would not say

I was that great, I was just quite tough. [Team-C]

Reaction to specific incidents was also less consistent and often less positive.

Well the sort of 10 to sort of 17, 18 years should be a natural yearly progression. But because I, because I broke my arm, I wouldn’t say I didn’t improve but I just stood still. Well I’d say I didn’t improve, I just sort of stood still for well, 18 months. And it was an issue because when my arm got fixed I hadn’t grown, and everyone else seemed to have grown. [Team-C]

Once again in contrast, As’ early careers appeared “blessed” (participant’s term) due to the lack of challenge and the perceived easiness of progression.

I just went from year to year, stage to stage and everything was great. So many people interested in me, talking to me about what it would be like to play premiership. The dream seemed really real. [Team-A]

Things came so easily to me…the skills, techniques, tactics…I felt no pressure and really agreed with when everyone told me ‘you’re a natural.’ [Individual-A]

Unfortunately, for all of the As, there came a moment of reckoning, which usually seemed unexpected. For example, one of the individual sport As described how:

The previous year had gone so well…national squad selection, lots of support, then the Winter of 2006 everything just blew up. I was suddenly lost…I didn’t know where to turn and the support just seemed to evaporate. [Individual-A]

Some of these derailments occurred because of injury and perceived insufficient or overly slow recovery. Notably, and in contrast to their more successful counterparts, As seemed to attribute externally in relation to the challenge and how they failed to cope.

By the time I got back, everyone seemed to have kicked on. I tried for a bit but I just couldn’t get back. I seemed to have lost my mojo. [Combat- A]

I sort of lost enthusiasm for it because I did not feel like it was – I almost felt let down that I had, especially before the second operation… why was my injury different from anyone else’s, how come mine had to be 14 months for the same surgery that someone else had done for 3 months. [Team-A]

#### Reflection and Reward

Clear differences were apparent in how different categories of participant thought about their sport and also in how they perceived progress and, consequently, administered self-reward. For example, the SCs seemed intrinsically driven, with their reflection sometimes at an almost spiritual level. The following quotations typify this:

I was a thinker after a game, or a competition. So I could analyze, I was good at analyzing myself. Both when I won and when I lost, I would think. [Team-SC]

I err, have had an inside commentator all my life. I could see myself doing things before I went to sleep… I would see myself scoring a goal. I daydreamed a lot, but always in situations where I succeeded. I lifted a lot of World Cup trophies in my imagination! [Team-SC]

The SCs described how this intrinsically focused attention to detail was a key part of their preparation:

After every event and training session, *every* [participant’s emphasis), I would complete my diary, highlighting areas for development and setting goals. Man was I anal! But I had to do it or I was pissed with myself all day. [Individual-SC]

For me it was all about getting better; about perfecting this combination, then this one. Building my armory so that I felt…so I would be impregnable. [Combat –SC]

Cs also described how they engaged in reflection, but this seemed more focused externally, dominated by comparisons with close rivals and competition results.

I was always scanning [sport magazine] for ranking lists, who had done what over the weekend. If I was ahead I was happy; if someone passed my SB I would fume all day. [CGS-C]

The Cs were very focused on external factors and this tended to drive their preparation as articulated by the following quotations:

Scores at the national ranking events were my focus…how am I doing against X or Y. If they seemed to be scoring better than me, I would consider doing what they did, even changing coaches. In fact, I did that twice on the way up. [Individual-C]

My whole focus was competition. I would beat most people in randori [free practice training fights] but it meant nothing…it had to be in the event. Looking back, I recognize how foolish I was in not trying to transfer from one to the other. At the time, they were just completely separate entities. [Combat-C]

As also displayed this external, results focus, but to a significantly higher degree as their careers neared the clutch point of final decisions on progression.

Early doors I just played…I would score every game, sometimes two or three but it didn’t really matter. As I got toward the final stages of the Youth Team, before they decided who to sign, I got a lot more interested in how I was doing against the others in the team. [Team-A]

I didn’t really think about my performance…others did it for me. My coach, agent, girlfriends, Mum and Dad, they would all tell me how well I had played. [Team-A]

I was like that black guy in the Bruce Lee film…I was too busy looking good! Then it seemed like, all of a sudden, things got serious and difficult. [Combat-A]

The implications of this approach were particularly apparent for the As during key transitional challenges such as moving into the senior ranks of competition, as reported by this participant:

My thinking definitely changed when I became a senior. All of a sudden I wasn’t winning, not even placing really. I changed my thinking but also my new coach seemed a lot less supportive and positive and a lot more demanding. I was struggling to find the answers. [Individual-A]

#### The Role of Coaches and Significant Others in this Process

All categories of participants referred to the role of significant others in their progression. Once again, however, there were clear qualitative differences apparent between categories. SCs were mostly characterized by positive facilitation and gentle encouragement; interestingly, siblings played a significant role for many as exemplified in the following quotations:

It was when my older brother became professional and that opened the door in my mind that it was possible. [Team-SC]

The drive…it’s not from my parents. They were supportive, but they didn’t drive me, they didn’t push at all. But the brothers, we were really competitive to each other. [Team-SC]

All through my development from 13 to 18 being encouraged to compete with them I remember my older brothers kicking high balls and we would see who could catch them. [Team-SC]

Interestingly, the SCs described how parents took a back seat, and though interested, were not a significant driver of their development. One of the CGS SCs described how:

[my parents were] not really pushy, it was kind of just gentle encouragement. They didn’t get, you know some parents get really involved? They were never really involved, they’d just come and watch me, support me. But they never wanted to know what I was doing training wise and they never really got involved in that way, and that helped. [CGS-SC]

The SCs reported that this behavior on the part of significant others have important implications on their development. For example, participants described how:

I learnt how to be very self-sufficient at the time. It’s not that they [my parents] didn’t want to do it, they just didn’t need to do it, I could do it myself. [Individual-SC]

As such, although parents were described as being a supportive influence, the SCs reported that they did not have a significant coaching role:

He was always there on a Sunday morning. He would go to some County games and pretty much I think even now he comes across country to watch me play, so he has probably been to 95% of the games I’ve played, so he’s clearly big in support but he has never played the game or been on the coaching side of things, but he has given huge support. [Team-SC]

Interestingly, SC’s coaches also mostly seemed to take a chilled, longer term perspective, often in contrast to the performer’s immediate ambitions.

I think [coaches name] was great in the fact that he never wanted to rush anything where as I always did. I wanted to be better, and I wanted to start winning things straight away. He always had in his mind that it was a long journey. And that’s the sort of thing that worked so well, he developed me as an athlete really slowly so I would always achieve the things I wanted to achieve later on in my career. [CGS-SC]

X was just what I needed. Always offering me a dose of realism…building me up after disasters, drawing my attention to weaknesses after big wins. Always friendly but always honest. [Combat –SC]

For Cs, involvement from parents seemed to be a bit more hands on.

My Dad managed the Sunday league team so he was supportive. I wouldn’t say I got any special treatment, but I wouldn’t say he went the other way and kind of subbed me anymore than normal players. [Team-C]

He would come and coach me every Sunday, so he had a big impact and he still does now. He is still the first person I speak to after every single game and it’s kind of like a bit of a personal coach for me. [Team-C]

Several Cs highlighted how new relationships seemed to offer useful impetus for change.

When I met my wife it put me back on track a bit… it coincided with me doing well. [Team-C]

Meeting and moving in with X was the making of me. She encouraged me to re-evaluate my life, what part [sport] could play in my life and ours. [Individual-C]

Coach relationships seemed slightly more pushy for the Cs but also changed more frequently.

X was always wanting to dissect my performance. He was very intense and, as I got older, it really started to antagonize me. [Individual-C]

I became increasingly aware in that middle stage that coaches wanted to ride me to the top. I spent a lot of time chopping and changing, trying to find someone who would look after me instead of the other way around. [CGS-C]

For the A category, significant others appeared to play a big (sometimes perceived as too big) part in their sport.

My parents, Dad especially was always there…shouting instructions from the touchline, pushing me to practice at home. Really, I just wanted to be out with my mates, even though we would still be kicking a ball around. I felt like [sport] stole my childhood. [Team-A]

The As described how parents were an ever-present factor that was always facilitative of good performances:

Mum and Dad would drive me everywhere, watch with much nail biting (I was always aware of their nerves) then drive me home with a big inquest. I sometimes envied my younger sister who didn’t have all this shit. [Individual-A]

Coaches also seemed to be more demanding, often seeming to drive the bus more than the performer.

X was the driving force. When I was younger, he would collect me from home, drive me to the club, train me then drive me back…talking about [sport] all the way. Let me tell you it was f∗∗∗∗∗ intense. [Combat-A]

Coach became more like a father than my father…a domineering one sometimes but very focused on how I was behaving, what I was eating, boyfriends, etc. [CGS-A]

Interestingly, however, the loss of such an authority figure as progress floundered was a problem for several.

You go from a stage of having a conditioning coach that stretches you … you know what you are doing each week, to going to this senior squad and you are not really sure … some days you are on loan to [Club] and then some days everyone’s been pulled from [Club] and then you are training again and it’s so wishy washy you don’t really know where you stand. [Team-A]

The As noted how they were ill-prepared to cope with these changes:

It was a real feeling of release to get away from [Coach/Father] and go to University. But once there I seemed to lose my way. No-one telling me what to do…I just lost interest. [CGS-A]

As in the previous section, an intrinsic-extrinsic difference was apparent across the categories.

### Non-Discriminating Factors

#### Incidence and Types of Trauma

Reflecting the stated purposes of this investigation and against the backdrop for the recent trend for trauma-focused investigations, we were particularly keen to examine the incidence and impact of trauma across the different categories. These data are summarized in **Table 2**. This quantitative style may seem at odds with the tone and qualitative approach of the paper but, we would suggest, offer the clearest and most succinct way of presenting an accurate picture.

**Table 2:** Self-reported incidence of major and minor trauma (using categorization utilized by Howells and Fletcher (2015).

Type	Level/Reported impact	Self-presented (SP) OR After probing (AP)	SC	C	A
Developmental stressors (e.g., developmental disorders)	High	SP	0	0	2
		AP	0	0	+1
	Low	SP	1	1	1
		AP	+1	+1	+2
External stressors (e.g., family dysfunction)	High	SP	1	1	2
		AP	+1	+2	+1
	Low	SP	0	1	1
		AP	+1	0	+2
Embodied states (e.g., injury/illness)	High	SP	5	6	7
		AP	+2	+1	+4
	Low	SP	2	2	3
		AP	+2	+1	+3
Psychological states (e.g., body dissatisfaction, depression)	High	SP	2	1	4
		AP	0	+1	+2
	Low	SP	2	2	3
		AP	+1	0	0
Externalized behaviors (e.g., self-harm, substance abuse)	High	SP	0	0	2
		AP	0	0	0
	Low	SP	1	2	3
		AP	0	0	+2


*Numbers in each category are from a total of 18 participants. Plus signs show additional participants reporting this factor after probing.*

The numbers reflect the number of participants (out of 18 in each category of SC, C, and A) who spontaneously reported traumatic experiences, with the plus sign numbers in the AP (after probing) rows representing additional participants who reported trauma only as a result of questioning. Following the description of the incident or occurrence, participants were asked to rate the impact of the trauma on them personally, using a simple high–low scale of perceived impact. Subsequently, and as a part of the analysis process, participant descriptions were classified into different types, using the same classification apparent in the recent paper by Howells and Fletcher (2015). Whether spontaneously reported, or identified after probing, incidents or occurrences of trauma described were dealt with in a variety of ways. Of most relevance, both the reporting and rating of the issue was a matter of perception, with each participant relating the impact to how well s/he had coped with it. Thus, for example, a very similar incident (illness with glandular fever) was spontaneously reported and rated as a high impact by four As but rated low by two SCs. Of course, we have no way of balancing the severity of the incident; our point is, however, that it is the perceived impact, determined in no small part by how well the participant coped with the challenge, which seems to be a factor.

The clear picture to emerge is a lack of universal trauma in the development pathways of performers across all levels. Indeed, if anything, there seems to be a higher incidence of such trauma in lower rather than higher achievers. Furthermore, with the notable exception of sport related challenges (as described in the previous sections), these challenges seem not to be associated with either specific growth (cf. Fletcher and Sarkar, 2012) or incidence of significant sporting success.

## Discussion

Our suggestion would be that, on first review, our discriminating factors offer little new but rather, confirm numerous prior studies which, in simple terms, show that super champs can be differentiated from their less successful counterparts on a variety of factors. On deeper examination, however, several important distinguishing characteristics emerge. As an example, consider the ways in which participants conceptualized, thought about, and actioned their experiences. The importance of finding meaning was clear, especially in the higher achievers (cf. Linley and Joseph, 2004, 2011). With certain notable exceptions, however, these reflections seemed to be proactively driven. In other words, excepting specific issues, SCs and, to a lesser extent Cs, appeared to arrive at a challenge with an established attitude and approach whilst As seemed almost entirely reactive. We suggest that the facilitative rather than directive styles of parenting and coaching experienced by most SCs may have been a contributory factor to this characteristic; such opinions were explicitly expressed by several SCs. The point is that high achievers seem to hold a positive proactive coping and “learn from it” approach to challenge (cf. Rosenbaum, 1983 and, perhaps, more recent ideas on Mental Toughness) even *before* they get started on the rocky road. We would suggest the age of 13 as an important watershed in this regard. The important next step is clearly for longitudinal study, and we currently have two projects underway to meet this need.

Of course, this is not to say that participants didn’t learn from challenges (trauma) encountered on the pathway. Notably, however, all of the SCs’ learning and development seemed to be associated with *sporting* challenges; most commonly injury but also de-selection or non-selection at crucial times. Once again by contrast, lower achievers were characterized by external attributions and often seemed almost surprised by failure. Such findings carry significance for the provision and periodization of challenge on the pathway, supporting the idea of speed bumps and phases of reflection/regeneration/growth as athletes develop (MacNamara and Collins, 2014).

In parallel to these findings and, of significance for current research directions, we would highlight the lack of discrimination across categories associated with the incidence of trauma and, more particularly, life or non-sport trauma. Notably, and in substantial contrast to some recent approaches (e.g., Sarkar et al., 2014), **Table 2** shows that, in our sample at least, there were no apparent differences in the incidence of trauma, either self-reported or as revealed by probing, between high and low achievers. There are several explanations for this. Based on these data, and previous research (Tedeschi and Calhoun, 1995; Nicholls et al., 2006), we would suggest that higher level performers are established in proactive coping habits (Greenglass and Fiksenbaum, 2009), to the extent that they just don’t acknowledge or experience trauma as traumatic as others; once again, this is supportive of a qualitative/perceptual difference instead of a quantitative “how much trauma” distinction. We would suggest that future studies must consider comparisons *between* levels of achievement, rather than just listing the self-reported incidence of challenge for established champions. The notable exception is *sporting* challenge which (perhaps relating to its importance in their identity and self-schemata – Brewer et al., 1993; Bianco et al., 1999; Nasco and Webb, 2006) were particularly formative for SCs. In this regard, we should highlight that many of the trauma studies fail to discriminate between sporting and life challenges. For example, Sarkar et al. (2014) report (positively in our view) mostly on sporting challenge although other papers (e.g., Fletcher and Sarkar, 2012; Howells and Fletcher, 2015) seem more driven toward life trauma.

Another focus relates to the quantity of traumatic experience, with research pointing toward moderate levels of challenge being associated with more positive outcomes (Seery et al., 2010). Once again, we would suggest that the differences between performers may be more qualitative, in that the positive and proactive coping/optimism blend which characterizes high achievers makes them frame challenges as more moderate and/or controllable than is the case for others. This is certainly the case with the larger populations of SCs (currently > 70) which form the rest of our sample. Matched As are the difficult ones to recruit!

Extending from this quantitative/qualitative balance, it is noteworthy that there can often be low correlation between perceived and actual growth following trauma (Frazier et al., 2009; Gunty et al., 2011). Indeed, perceived growth may well be a feature of the individual’s coping strategy (it *hasn’t* killed me and I *am* stronger!). As Joseph et al. (2012, p. 319) observe,

It may be that perceptions of growth are at times illusory and a way of coping with distress. As such, researchers need to be wary of taking reports of growth at face value, particularly in the immediate aftermath of a crisis when people are most distressed.

We would also like to comment on the inherent problems of self-presentational bias (cf. Arkin et al., 1980) which may combine with these considerations to call into question self-reported, after the fact interpretations of trauma impact. It seems to us that this could be a particular issue in autobiographies, especially if these are ghost written in partnership with the performer. Indeed, one of our Team SC participants made his feelings clear on this: “F******* sports books…people telling their stories to make themselves look good.” Another Team SC questioned the veracity of autobiographical reports as follows “I’ve lived through the competitions they are writing about and I certainly didn’t see what they saw!” In this regard, autobiographical sources offer an *after the fact* interpretation rather than *this happened and led to this* description: “autobiographies emphasize not facts, but personal experiences and personal lives as cultural constructions” (Stewart et al., 2011, p. 583). This is in no way to question the veracity but rather to stress the need to consider such sources as personal interpretations: very useful for certain purposes [e.g., the meaning of injury as in Stewart et al. (2011)] but perhaps less so for guiding the design of TD environments!

In summary, we feel that the differences between different levels of adult achievement relate more to what performers *bring to* the challenges than *what* they experience. This skills-based focus is completely in keeping with our PCDE approach and, whilst we completely acknowledge the importance of mindset, grit or resilience as parts of this skill set, we feel that the exclusivity inferred by some authors (in fairness sometimes second hand accounts rather than the researchers themselves) need further investigation and, if appropriate, justification. For the present, we suggest that a periodized and progressive set of challenge, preceded and associated with specific skill development, offers the best pathway to success for the majority.

## Author Contributions

All authors contribute equally to this manuscript.

## Conflict of Interest Statement

The authors declare that the research was conducted in the absence of any commercial or financial relationships that could be construed as a potential conflict of interest.
